# Aripiprazole LAI two‐injection start in a 16 year‐old adolescent with schizophrenia

**DOI:** 10.1002/npr2.12240

**Published:** 2022-02-19

**Authors:** Virginio Salvi, Cristina Appignanesi, Brodinela Marpepa, Laura Orsolini, Umberto Volpe

**Affiliations:** ^1^ 9294 Department of Clinical Neurosciences/DIMSC Università Politecnica delle Marche Ancona Italy

**Keywords:** adolescents, antipsychotics, aripiprazole, schizophrenia

## Abstract

**Background:**

Aripiprazole long‐acting injection (LAI) is approved for the treatment of schizophrenia in adults. Recently, Europe and Canada approved the use of the two‐injection start regimen: two separate injections of 400‐mg long‐acting aripiprazole along with a single 20‐mg dose of oral aripiprazole. Aripiprazole showed efficacy in the treatment of adolescents with acute schizophrenia in several controlled trials, leading to its approval for 13‐ to 17‐year‐old adolescents with schizophrenia by the EMA. However, the LAI formulation still remains off‐label in adolescents.

**Case presentation:**

The patient was admitted to the General Psychiatry inpatient unit with a Positive and Negative Syndrome Scale (PANSS) total score of 136 and the Clinical Global Impression (CGI) score of 7. Aripiprazole was started and up‐titrated to 30 mg/d. After 3 weeks, the positive symptoms were significantly reduced; however, he still showed prominent negative symptoms. Clozapine 100 mg/d was added, and in the following 2 weeks, the patient appeared slightly more communicative and generally more aware of himself and the others. The PANSS total score decreased to 81. Due to poor insight, the patient was at‐risk not to take medications upon returning home; therefore, aripiprazole LAI was proposed. Since he urged to be discharged from the hospital, we opted for the two‐injection start. The medication was optimally tolerated, with no evidence of akathisia or other side effects. One month later, global functioning and illness insight improved; Positive and Negative Syndrome Scale score was 43 and CGI score 2.

**Conclusions:**

Aripiprazole LAI showed good efficacy and tolerability in an adolescent with schizophrenia. The two‐injection start regimen was a safe and effective option.

## Abstract 2

Although adherence to treatment is an issue in adolescents with schizophrenia, long‐acting injection (LAI) antipsychotics are not approved for use in this patient population. The article reports a case of a 16‐year‐old adolescent with schizophrenia treated with LAI aripiprazole two‐injection start regimen. The treatment was effective and well tolerated.

## 1 INTRODUCTION

Several evidence highlighted that schizophrenia should be treated as soon as possible in order to limit the detrimental effect of the untreated psychosis on the brain. Indeed, a longer duration of untreated psychosis (DUP) may impact the brain and its functioning through dopaminergic hyperactivity, prolonged hypothalamic‐pituitary‐adrenal activation, and persistent activity of catecholamines.^1^ Plus, a longer DUP has been associated with reduced functional connectivity in the default, salience, and executive networks in youths with schizophrenia.^2^ Recently, a meta‐analysis of mainly prospective studies translated these observations into the clinic finding that a longer DUP correlates with more severe positive and negative symptoms and a lesser chance of remission at follow‐up.^3^ One of the most critical issues in the early phases of treatment is adherence, especially in adolescents and young adults, where comorbid substance abuse and lack of insight are more common.^4^ For this reason, the use of long‐acting injectable (LAI) antipsychotics has been advocated in early‐onset psychosis.^5^, ^6^ Furthermore, the use of LAI antipsychotics may carry other benefits, such as more consistent bioavailability, reduced peak‐to‐trough plasma levels, and improved patient and physician satisfaction.^7^

Aripiprazole LAI is approved for the treatment of schizophrenia in adults. Recently, Europe and Canada approved the use of the two‐injection start regimen, which consists in administering two separate injections of 400‐mg long‐acting aripiprazole along with a single 20‐mg dose of oral aripiprazole. This start regimen has the clear advantage of not requiring oral aripiprazole supplementation, eventually bypassing the potential issue of lack of adherence to oral treatment. Aripiprazole showed efficacy in the treatment of adolescents with acute schizophrenia in several double‐blind placebo‐controlled studies,^8^, ^9^, ^10^ leading to its approval for 13‐ to 17‐year‐old adolescents with schizophrenia. However, the LAI formulation still remains off‐label in youths.

## 2 CASE PRESENTATION

Here, we describe the case of a 16‐year‐old adolescent successfully treated with aripiprazole LAI started with the two‐injection regimen.

The patient had a history of social withdrawal since age 13, when parents reported his disengagement from school and friends. At age 14, he started being aggressive toward objects at home, out of flimsy triggering events. In one of those situations, he was carried to the emergency department of the local Children's Hospital and eventually admitted to the Child and Adolescent Psychiatry inpatient unit, where he was administered aripiprazole 5 mg/d. In the following weeks, his tantrums remitted, yet he continued to being socially withdrawn. After a month, aripiprazole was unduly discontinued, and he returned to be aggressive. He was eventually readmitted to the Children's Hospital, where lithium and olanzapine were initiated. This time, the pharmacological treatment seemed ineffective; moreover, the patient gained 10 kg in the ensuing 3 months, which led to the discontinuation of the medications.

Following treatment interruption, he showed good functioning during summertime, even hanging out with few friends at the seaside. However, in autumn, he became again socially withdrawn and started conflicting with his parents upon minimal triggers. In the 2 months preceding the index hospitalization, the patient spent all days listening at trap music, muttering and laughing to self, oftentimes showing perplexity. He did not attend school anymore and, whenever his parents confronted him, he would suddenly break down yelling nonsense and being aggressive toward objects of the house.

In May 2021, he was brought to the Emergency Department and admitted to the General Psychiatry inpatient unit. At hospital admission, the patient looked agitated and perplexed, showing motor mannerisms such as odd postures, and in some moments talking with his auditory hallucinations, his thought fragmented and disorganized. His Positive and Negative Syndrome Scale (PANSS)^11^ total score was 136 and the Clinical Global Impression (CGI)^12^ score 7 (Table 1). The Structured Clinical Interview for DSM‐5 (SCID‐5)^13^ was administered, and the patient diagnosed with schizophrenia. Aripiprazole was immediately started and up‐titrated to 30 mg/d in 1 week. After 3 weeks, the positive symptoms were significantly reduced. The patient appeared more in contact with doctors and nurses, being able to sustain brief conversations, and his auditory hallucinations significantly reduced. On the flipside, he showed minimal to absent speech, no interactions with others, and a remarkably flat affect. At this time, the PANSS total score was 101. The resistance to treatment of the negative symptoms, together with the previous nonresponse to another antipsychotic (ie, olanzapine), prompted us to add clozapine up to 100 mg/d. In the following 2 weeks, the patient appeared gradually more relaxed and slightly communicative, smiling a few times and generally more aware of himself and the others. The PANSS total score further decreased to 81.

**TABLE 1:** Rating scale scores across time points

	Hospital admission	Week 3	Week 5	Week 10
PANSS positive	27	15	11	7
PANSS negative	46	39	30	14
PANSS general	63	47	40	22
PANSS total	136	101	81	43
Clinical Global Impression	7	6	4	2

Abbreviation: PANSS, Positive and Negative Syndrome Scale.

However, insight was still poor and he started saying that, since he felt better, medications could be wiped off. His parents were concerned that he would not take the meds upon returning home; therefore, we proposed to start LAI aripiprazole. Having highlighted the benefits of a stable antipsychotic regimen, his parents and, eventually, the patient himself accepted the proposal and signed an off‐label medications informed consent.

Since his conditions were stable and he urged to be discharged from the hospital, we opted for the two‐injection start. The medication was optimally tolerated, with no evidence of akathisia or other side effects. His conditions remained stable, and he could safely be discharged the day following the injection. Soon after discharge, outreach community treatment was deployed, with professional educators working with the patient on problem solving and social skills, plus monthly psychiatric consultations. One month later, global functioning and illness insight improved. The positive symptoms were no longer present, affect was better modulated, and the patient started being engaged in social contacts again. The PANSS total score was 43 and CGI score 2.

## 3 DISCUSSION AND CONCLUSIONS

Literature on the use of LAI antipsychotics in adolescence is extremely scarce. The only published study reported similar efficacy for aripiprazole, paliperidone, and risperidone LAI in a small group of thirty 16‐year‐old adolescents with schizophrenia after 2‐3 weeks of open‐label treatment.^14^ Being lack of insight and noncompliance to treatment a major issue in adolescents with schizophrenia, this dearth of studies is problematic.

Aripiprazole LAI requires oral supplementation in the first 2 weeks of treatment, which may expose the noncompliant patient to a higher risk of relapse. For this reason, the two‐injection start regimen may be an interesting option, also helping to reduce the duration of hospital stay allowing an earlier discharge. Here, we showed, for the first time, that the two‐injection start regimen of aripiprazole LAI was a safe and effective option in a 16‐year‐old adolescent with schizophrenia. Further research on the efficacy and safety of LAI antipsychotics in adolescence is very much needed. Plus, the putative protective effect of continuous treatment with LAI antipsychotics on the adolescent brain should be explored in future studies.

## CONFLICT OF INTEREST

The authors declare no conflict of interest.

## AUTHOR CONTRIBUTIONS

VS conceptualized and wrote the manuscript. CA and MB did the clinical evaluation and rating scale administration. VS, LO, and UV were involved in the literature search and revisions. All authors read and approved the final manuscript.

## APPROVAL OF THE RESEARCH PROTOCOL BY AN INSTITUTIONAL REVIEWER BOARD

The ethics committee is not required to review case reports.

## INFORMED CONSENT

The patient has consented in a written form to the submission of the case report for submission to the journal.

## Data Availability

Data sharing is not applicable to this article as no new data were created or analyzed in this study.
